# Establishment of a Murine Graft-versus-Myeloma Model Using Allogeneic Stem Cell Transplantation

**DOI:** 10.1371/journal.pone.0113764

**Published:** 2014-11-21

**Authors:** Marilène Binsfeld, Yves Beguin, Ludovic Belle, Eléonore Otjacques, Muriel Hannon, Alexandra Briquet, Roy Heusschen, Pierre Drion, Jenny Zilberberg, Bjarne Bogen, Frédéric Baron, Jo Caers

**Affiliations:** 1 Laboratory of Hematology, GIGA-Research, University of Liège, Liège, Belgium; 2 University of Liège, Liège, Belgium; 3 John Theurer Cancer Center, Hackensack University Medical Center, Hackensack, New Jersey, United States of America; 4 Centre for Immune Regulation, Institute of Immunology, University of Oslo and Oslo University Hospital, Oslo, Norway; 5 KG Jebsen centre for research on influenza vaccines, Institute of Immunology, University of Oslo and Oslo University Hospital, Oslo, Norway; Shanghai Jiao Tong University School of Medicine, China

## Abstract

**Background:**

Multiple myeloma (MM) is a malignant plasma cell disorder with poor long-term survival and high recurrence rates. Despite evidence of graft-versus-myeloma (GvM) effects, the use of allogeneic hematopoietic stem cell transplantation (allo-SCT) remains controversial in MM. In the current study, we investigated the anti-myeloma effects of allo-SCT from B10.D2 mice into MHC-matched myeloma-bearing Balb/cJ mice, with concomitant development of chronic graft-versus-host disease (GvHD).

**Methods and results:**

Balb/cJ mice were injected intravenously with luciferase-transfected MOPC315.BM cells, and received an allogeneic (B10.D2 donor) or autologous (Balb/cJ donor) transplant 30 days later. We observed a GvM effect in 94% of the allogeneic transplanted mice, as the luciferase signal completely disappeared after transplantation, whereas all the autologous transplanted mice showed myeloma progression. Lower serum paraprotein levels and lower myeloma infiltration in bone marrow and spleen in the allogeneic setting confirmed the observed GvM effect. In addition, the treated mice also displayed chronic GvHD symptoms. *In vivo* and *in vitro* data suggested the involvement of effector memory CD4 and CD8 T cells associated with the GvM response. The essential role of CD8 T cells was demonstrated *in vivo* where CD8 T-cell depletion of the graft resulted in reduced GvM effects. Finally, TCR Vβ spectratyping analysis identified Vβ families within CD4 and CD8 T cells, which were associated with both GvM effects and GvHD, whereas other Vβ families within CD4 T cells were associated exclusively with either GvM or GvHD responses.

**Conclusions:**

We successfully established an immunocompetent murine model of graft-versus-myeloma. This is the first murine GvM model using immunocompetent mice that develop MM which closely resembles human MM disease and that are treated after disease establishment with an allo-SCT. Importantly, using TCR Vβ spectratyping, we also demonstrated the presence of GvM unique responses potentially associated with the curative capacity of this immunotherapeutic approach.

## Introduction

Multiple myeloma (MM) is a malignant plasma cell disorder that accounts for approximately 10% of all hematological cancers [1]. Despite recent advances, long-term survival is rare after autologous stem cell transplantation and/or treatment with recently introduced anti-myeloma agents, and disease recurs in virtually all patients. Therefore, other therapeutic approaches need to be developed to complement the current strategies. Several immune alterations have been described in MM patients. These alterations are caused in part by the replacement of normal bone marrow with malignant plasma cells, suppressing normal hematopoiesis. Moreover, the immune response is directly suppressed by MM cells and through their interactions with the microenvironment [2]. As the immune response impairment contributes to MM progression, cellular immunotherapy appears to be a promising therapeutic approach.

Allogeneic stem cell transplantation (allo-SCT) is a form of cellular immunotherapy that is widely used to treat hematological malignancies [3]. Much of the curative potential of allografts is attributed to the “graft-versus-tumor” (GvT) effect [4]. In MM, evidence for a graft-versus-myeloma (GvM) effect was provided by the ability of donor lymphocyte infusions to induce complete responses in patients who initially relapsed after allo-SCT [5], and by the association between chronic graft-versus-host disease (GvHD) and a decreased incidence of relapse after transplantation [6], [7]. However, despite evidence of GvM effects, allo-SCT has remained a controversial treatment modality in MM [8], [9]. Given the high relapse rate of MM after allo-SCT [7], some of the current clinical trials focus on combining non-myeloablative allo-SCT with new drugs given for post-transplantation maintenance therapy [10]. However, the introduction of immunomodulating agents that could improve GvT effects may inadvertently induce GvHD. This is well illustrated in a recent study by the HOVON group, where lenalidomide maintenance after non-myeloablative allo-SCT increased acute GvHD, and strongly suggests that new therapies aimed at modulating GvM effects should ideally be tested first in animal models [11].

Mouse models have contributed to the understanding of MM biology and to the introduction of novel agents [12], and are of great interest in the preclinical evaluation of cellular immunotherapy [2]. Currently, only two immunocompetent murine models have been described in which allo-SCT is associated with a GvM effect [13], [14], but these models do not resemble human MM disease [13] or do not use allo-SCT as a curative treatment for established disease [14]. So far, an immunocompetent murine GvM model in which allo-SCT is used for the treatment of established MM that resembles human disease, marked by bone marrow tropism and osteolytic lesions, has not been described.

In the current study, we investigated the anti-myeloma effects of allo-SCT from B10.D2 mice into myeloma-bearing Balb/cJ mice (H-2^d^ MHC-identical, but differing at minor histocompatibility loci) which results in sclerodermatous chronic GvHD [15], [16]. Myeloma-bearing Balb/cJ mice were inoculated with the myeloma cell line MOPC315.BM, originating from Balb/c mice [17], that presents bone marrow tropism [18].

## Materials and Methods

### Ethical statement

All experimental procedures and protocols used in this investigation were reviewed and approved by the Institutional Animal Care and Use Ethics Committee of the University of Liège (Belgium), reference 1016. The “Guide for the Care and Use of Laboratory Animals” [19], prepared by the Institute of Laboratory Animal Resources, National Research Council, and published by the National Academy Press, was followed carefully as well as European and local legislation. Animal welfare was assessed at least once per day, and all efforts were made to strictly control animal suffering during the experiments (e.g. development of a decisional system to follow the mice, application of humane endpoints with precise response to specific symptoms including use of dietary supplements, analgesic administration and sacrifice).

### Animals

Balb/cJ (H-2^d^) and B10.D2 (H-2^d^) mice were purchased from Jackson Laboratory (Bar Harbor, ME, USA). Strains were kept and bred at the animal facility of our institute. Mice were used when they were between 10- to 14-wk-old.

### Myeloma cell line and model

The selection of the MOPC315.BM cell line, which is derived from the mineral oil-induced plasmacytoma cell line MOPC315 [17], [20], was previously described [18], [21]. The parental MOPC315.BM cells and the firefly luciferase transfected cells (MOPC315.BM.Luc) were provided by Prof. Bjarne Bogen [18]. Luciferase-transfected MOPC315.BM cells were used for all experiments. Cells were maintained in culture at 37°C in 5% CO_2_ using a RPMI 1640 medium (Sigma-Aldrich, Bornem, Belgium) containing 10% fetal bovine serum (FBS) (Sigma-Aldrich) and 1% Penicillin (100 U/ml)/Streptomycin (0.1 mg/ml) (Sigma-Aldrich).

Intravenous (i.v.) injection of MOPC315.BM cells results in tumor development with a restricted localisation in the bone marrow and spleen and is associated with osteolytic lesions, validating the model as a multiple myeloma model. In advanced disease stages, bone marrow infiltration can cause paraplegia in mice through spinal cord compression [18]. Mice injected with MOPC315.BM cells were monitored daily for general condition and locomotion. They were sacrificed when presenting locomotion trouble/paraplegia, deterioration of general condition or apathy. Animals that were not immediately sacrificed when presenting locomotion trouble/paraplegia, e.g. because they were receiving allo-SCT treatment, received analgesic administration (buprenorphine 0.05 mg/kg twice per day) and were very closely monitored for general condition and activity.

### Graft cell suspension

Spleens and bone marrows (femurs and tibias) from donor mice were harvested and homogenized in RPMI 1640 medium containing 10% FBS and 1% Penicillin/Streptomycin ( = complete medium). Red blood cells were lysed using sterile filtered RBC lysis buffer (eBioscience, San Diego, USA) and cells were washed, resuspended in phosphate buffered saline (PBS) containing 3% FBS, and filtered through a 70 µM nylon membrane. For CD8 T-cell depletion, the “Mouse CD8α positive selection kit” (Stem Cell, Grenoble, France) was used according to the manufacturer's EASYSEP depletion protocol. Finally, cells were suspended in 200 µl PBS for i.v. injection.

### Bioluminescence measurement

Beetle luciferin (Promega, Leiden, Netherlands) solubilized in PBS was injected intra-peritoneally into mice (3 mg/mouse in 100 µl). Bioluminescence was measured within 10 to 20 minutes using VIVOVISION IVIS 200 (Xenogen, Alameda, USA). Results were analysed and quantified using Living Image software (Xenogen).

### *In vivo* experimental design

**Figure 1:**
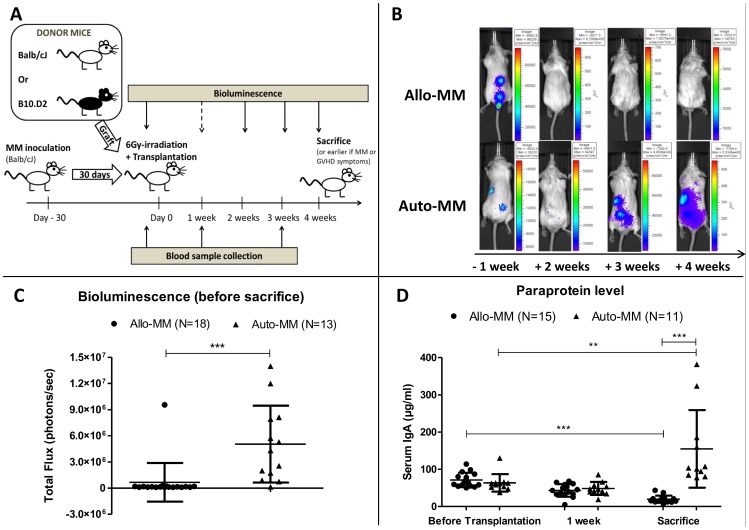
Graft-versus-myeloma effect. (**A**) Experimental design and monitoring. (**B**) Tumor burden. Representative examples of bioluminescence evolution (dorsal side) for Allo-MM (top row) and Auto-MM (bottom row) mice 1 week before transplantation, and 2, 3 and 4 weeks after transplantation. The mouse in the top row already displayed paraplegia before transplantation, and completely recovered after transplantation. (**C**) Bioluminescence quantitation. Total flux (photons/sec) measured on the dorsal side just before sacrifice (mean±SD), ***p<0.0001 (Mann-Whitney test). (**D**) Paraprotein level. Serum IgA quantitation (µg/ml) by ELISA before transplantation, 1 week after and at sacrifice (mean±SD),**p = 0.0005; ***p<0.0001 (Mann-Whitney test).

Balb/cJ recipient mice were injected intravenously with 2.5×10^5^ MOPC315.BM.Luc cells. MM development was allowed to proceed for 30 days, and monitored by bioluminescence studies. At day 30 post-inoculation, mice were irradiated with 6 Gy (Total Body Irradiation) from a ^137^Cs source (GammaCell 40, Nordion, Ontario, Canada). After 6 hours, mice were transplanted by i.v. injection of 1×10^7^ bone marrow cells and 7×10^7^ splenocytes from donor mice [allogeneic: B10.D2 donor; autologous: Balb/cJ donor]. Myeloma-bearing mice that received allogeneic or autologous transplant are referred to as “Allo-MM” or “Auto-MM” mice, respectively. Mice were sacrificed after appearance of myeloma symptoms (e.g. paraplegia), GvHD symptoms or apathy. Bioluminescence monitoring allowed tracing of luciferase-transfected myeloma cells and assessment of tumor development. This transplantation protocol was adapted from Jaffee and Claman who developed a murine model of chronic GvHD [15]. Experimental and monitoring strategies are summarized in Figure 1 A.

### Donor sensitization experiments

For GvHD studies, healthy Balb/cJ recipient mice were irradiated and transplanted with B10.D2 grafts as described in “experimental design”. Before transplantation to Balb/cJ mice, B10.D2 donor mice were not sensitized (controls), or sensitized by i.v. injection of 5×10^5^ myeloma cells (MOPC-sensitized) or Balb/cJ splenocytes (Balb/c-sensitized) 21 days before sacrifice for graft harvesting. GvHD symptoms were evaluated with a scoring system adapted from Sakoda et al [22]. The score is based on weight loss (<10% = 0; 10–20% = 1; >20% = 2), “hunched-back” position (normal = 0; hunched-back while resting = 1; persistent = 2), activity (normal = 0, reduced activity = 1, apathy = 2), alopecia (normal = 0, <1 cm^2^ = 1, >1 cm^2^ = 2) and skin fibrosis (normal = 0, fibrosis = 1; scabs = 2) with a maximum score of 10. The animals' conditions were controlled daily, and the GvHD score was calculated at least 3 times per week. Mice were sacrificed at the latest at a score of 8/10 or when apathic.

### *In vitro* co-cultures

Cell suspensions were obtained from spleens as described earlier. Effector cells were obtained from spleens of Allo-MM mice, sacrificed 2 weeks after transplantation. Target cells (MOPC315.BM cells or Balb/cJ splenocytes) were irradiated with 50 Gy using a ^137^Cs source (GammaCell 40) and washed with complete medium after irradiation. The effector:target ratio was 20∶1 and cells were co-cultured for 5 days in 6-well plates using complete medium, containing 0.1% of Heparin (LEO Pharma, Lier, Belgium). Effector cells were harvested and analysed by flow cytometry at the end of the co-cultures.

### Flow cytometry

Cell suspensions were obtained from spleen, lymph nodes, bone marrow and blood. Red blood cells were lysed as described earlier. Extracellular staining was performed in PBS containing 3% FBS. Intra-cytoplasmic or intra-nuclear staining was performed using BD Cytofix/Cytoperm (BD Biosciences, San Diego, CA, USA) or Foxp3 Staining Buffer Set (eBioscience), respectively. Antibodies were incubated for 30 min at 4°C. The Streptavidin/PerCPCy5.5 complex and the following antibodies were purchased from eBioscience: anti-CD4/eFluor450 (RM4-5); anti-CD8/PECy7 (53-6.7); anti-CD49b/Biotin (DX5); anti-CD69/APC (H1.2F3); anti-B220/APCeFluor780 (RA3-6B2); anti-Foxp3/PE (FJK-16s); anti-CD44/APC (IM7); anti-CD62L/APCeFluor780 (MEL-14). The following antibodies were purchased from BD Biosciences: anti-CD229.1/FITC (30C7); anti-CD3e/v500 (500A2) or from Invitrogen: anti-IgA/FITC. A specific antibody directed against MOPC315.BM paraprotein (Ab2.1-4/Biotin) was kindly provided by Bjarne Bogen. Quantitation of blood cells was determined using BD Trucount tubes (BD Biosciences). Flow cytometric data were acquired using a BD FACSCanto II flow cytometer (BD Biosciences) and the BD FACS DIVA software, and analysed with the FlowJo software (Tree Star, Ashland, OR, USA).

### Serum paraprotein quantitation

Serum paraprotein levels were measured using an ELISA for mouse IgA (Mabtech, Sweden) according to the manufacturer's instructions and analysed with a Multiskan FC Plate reader (Thermo Scientific) with the SkanIt for Multiskan FC 3.1 software.

### TCR Vβ CDR3-size spectratype analysis

T cells were isolated from splenocytes of B10.D2 mice, i.e. “non-sensitized” (control mice), “MOPC-sensitized” or “Balb/cJ-sensitized” mice 21 days post-sensitization. CD8 T cells were isolated using the “Mouse CD8α positive selection kit”. The CD8-negative fraction was further depleted to eliminate residual CD8 cells and constituted the “CD4 fraction” (>80% of T cells were CD4^+^). Cell pellets were suspended in TriPure Isolation Reagent (Roche, Vilvoorde, Belgium). RNA extraction was performed using chloroform and isopropanol following the manufacturer's instructions. Isopropanol phase containing RNA was transferred on “RNeasy Mini kit“ columns (Qiagen, Venlo, Netherlands) and RNA extraction was finalized following the manufacturer's instructions. Genomic DNA was removed using recombinant RNase-free DNaseI (Roche, Vilvoorde, Belgium). cDNA was synthesized from RNA (2 µg) using oligo(dT)_18_ primers with the “Transcriptor First Strand cDNA synthesis kit” (Roche). Seminested PCR was performed using sense primers for a panel of murine Vβ families and two Cβ anti-sense primers, the second being fluorescently labelled (IDT Technologies, Leuven, Belgium), as previously described [23], [24]. All PCR reagents were purchased from Applied Biosystems (Life Technologies, Gent, Belgium). The fluorescently labelled PCR products were run together with GeneScan ROX 500 Size Standard (Applied Biosystems) on a “DNA Analyzer 3730“ (Applied Biosystems) capillary electrophoresis system at the GIGA-Research Genomics facility of the University of Liège. CDR3-size spectratype analysis was performed with GeneMapper version 4.0 Software (Applied Biosystems).

Experiments were repeated three times, and the mean area of the different peaks, representing different CDR3-size lengths, was calculated for each Vβ family. A CDR3-size length was considered skewed when the mean area under the peak was higher than the mean+3SD of the same peak in the control condition (non-sensitized B10.D2 mice), as previously described [25].

### Statistics

Statistical significance between groups was determined using Mann-Whitney tests. Survival curves were compared using the Log-Rank test (Mantel-Cox). These statistical tests were performed with the Prism Software (Graph Pad Software, San Diego, CA).

## Results

### Graft-versus-myeloma effect

The experimental design is illustrated in Figure 1A. Briefly, Balb/cJ recipient mice were injected intravenously with luciferase-transfected MOPC315.BM cells. After MM development during the first 30 days, mice were irradiated and transplanted by i.v. injection of bone marrow cells and splenocytes from donor mice (allogeneic: B10.D2 donor; autologous: Balb/cJ donor). Myeloma-bearing mice that received allogeneic or autologous transplantation are referred to as “Allo-MM” or “Auto-MM” mice, respectively.

MHC-matched allo-SCT in the MOPC315.BM myeloma model resulted in strong anti-myeloma effects. We observed complete bioluminescence disappearance in 17 out of 18 Allo-MM mice from 4 independent experiments (94%), whereas all 13 Auto-MM mice showed an initial decrease in bioluminescence (probably due to the irradiation) followed by increasing bioluminescence signals after transplantation and progressive myeloma disease (p<0.0001; Fig.1 B-C). Strikingly, two mice in the Allo-MM group already displayed paraplegia before transplantation and recovered completely. Serum paraprotein measurements demonstrated a significant decrease of paraprotein levels in the Allo-MM mice (p<0.0001). In contrast, paraprotein significantly increased in the Auto-MM group. Moreover, the Allo-MM group showed significantly lower paraprotein levels at sacrifice compared to the Auto-MM group (p<0.0001; Fig.1 D), as well as lower myeloma cell infiltration in the bone marrow (mean±SD: 0.03±0.04 vs. 1.3±1.3%; p<0.0001) and spleen (0.3±0.3 vs. 3.5±5.3%; p = 0.027). All together, these data demonstrate a potent GvM effect of allogeneic transplantation in this model. A similar anti-tumor effect was also observed when myeloma cells were injected subcutaneously, resulting in formation of solid tumors. After allo-SCT, solid tumors regressed into small residual tumors, whereas tumors in the autologous group continued to grow after transplantation (N = 6/group).

Regarding GvHD, 16 out of 18 mice in the Allo-MM group showed symptoms of chronic GvHD (alopecia, skin fibrosis, weight loss, “hunched-back” position, diarrhea) after day 21 post-transplantation, which is the time point for symptom appearance in the B10.D2→Balb/cJ GvHD model. The other 2 mice were sacrificed before day 21, one mouse due to myeloma progression and the other mouse due to a worsening of its general condition, probably due to transplant-related complications.

### Immune cell populations in the graft-versus-myeloma model

In order to determine which immune cells might be responsible for the observed GvM effect, we performed flow cytometry analyses on blood samples at different time points. At the time point of sacrifice also lymphoid organs (bone marrow, spleen, lymph nodes) were analysed.

**Figure 2:**
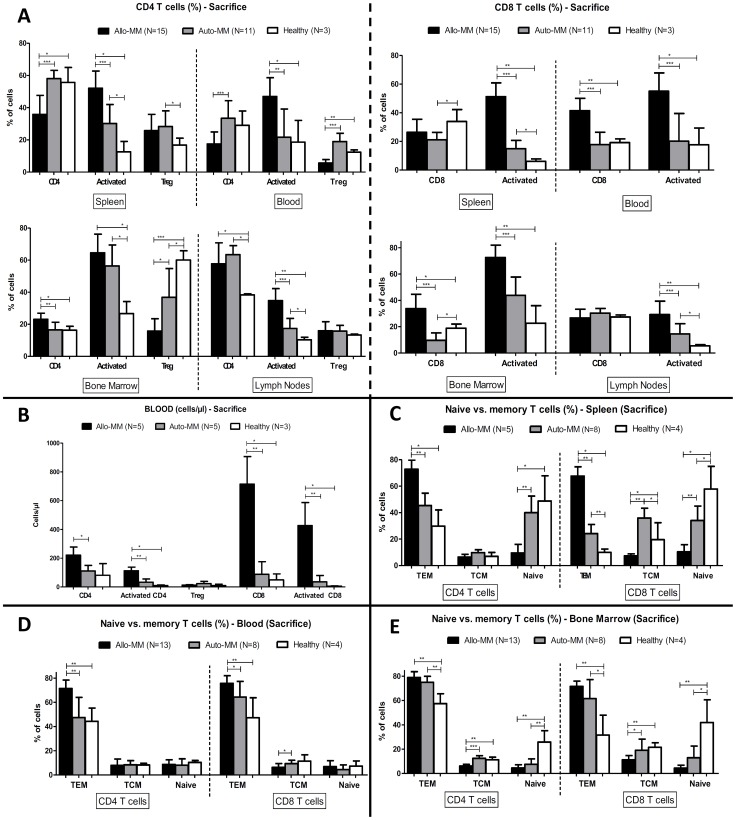
T-cell populations in graft-versus-myeloma model. Flow cytometry staining was performed for T cells on blood and lymphoid organs (spleen, bone marrow, lymph nodes) at sacrifice. (**A**) On the left, percentages (mean±SD) of CD4 T cells (within CD3^+^), activated CD4 T cells (CD69^+^ within CD4^+^) and regulatory T cells (Foxp3^+^ within CD4^+^) and, on the right, percentages of CD8 T cells (within CD3^+^) or activated CD8 cells (CD69^+^ within CD8^+^) in the Allo-MM or Auto-MM group, or in healthy Balb/cJ mice. *p<0.05; **p<0.01; ***p<0.001 (Mann-Whitney test). (**B**) T-cell quantitation in blood. Absolute cell numbers (mean±SD) per µl of blood at sacrifice are represented for CD4, activated CD4, regulatory CD4, CD8 and activated CD8 T cells in the Allo-MM or Auto-MM group, or in healthy Balb/cJ mice. *p<0.05; **p<0.01; ***p<0.001 (Mann-Whitney test). Naive vs. memory T-cell subsets in spleen (**C**), blood (**D**) and bone marrow (**E**). Percentages (mean±SD) of effector memory T cells (TEM, CD44^+^CD62L^−^), central memory T cells (TCM, CD44^+^CD62L^+^) and naive T cells (CD44^−^CD62L^+^) within CD4 or CD8 T cells at sacrifice are represented. *p<0.05; **p<0.01; ***p<0.001 (Mann-Whitney test).

At sacrifice, we observed significantly higher percentages of total CD8 T cells in the bone marrow and blood, and activated CD8 T cells in all investigated organs of Allo-MM mice compared to Auto-MM mice or healthy Balb/cJ mice (Fig.2 A). A large CD8 T-cell expansion (total and activated) was confirmed by absolute cell counts in blood at sacrifice (Fig.2 B). For activated CD4 T cells, an increase in percentages and absolute counts was also observed in Allo-MM mice at sacrifice, but this increase was much smaller compared to that of CD8 T cells (fold-increase in T-cell counts for Allo-MM vs. Auto-MM: CD8 T cells: 8.2x; activated CD8 T cells: 12x. CD4 T cells: 2x; activated CD4 T cells: 3.5x). In addition, percentages of regulatory T cells were significantly decreased in blood and bone marrow of Allo-MM mice compared to Auto-MM or healthy mice at sacrifice. Prior to sacrifice, kinetics in blood samples already showed higher total and activated T-cell counts (CD4 and CD8) 1 week after SCT in the Allo-MM compared to the Auto-MM group. Three weeks after SCT, activated CD8 were still significantly increased and total CD8 tended to be increased, whereas no increase in total or activated CD4 T-cell counts was present at this moment (data not shown).

Furthermore, we observed significantly higher percentages of effector memory subsets (CD44^+^CD62L^−^) within CD4 and CD8 T cells at sacrifice in the blood and spleen of the Allo-MM group, and the same trend was noted in the bone marrow (Fig.2 C-E). On the other hand, central memory (CD44^+^CD62L^+^) and naive (CD44^−^CD62L^+^) T-cell subsets were decreased in spleen and bone marrow of this group compared to the Auto-MM group or healthy mice.

Percentages and cell counts of NK (DX5^+^ CD3^−^) and NKT (DX5^+^ CD3^+^) cells did not differ between the two groups, whereas percentages of B cells (B220^+^ CD3^−^) in all lymphoid organs and blood were lower in Allo-MM mice compared to Auto-MM mice (data not shown).

### Involvement of CD8 T cells in the graft-versus-myeloma effect

**Figure 3:**
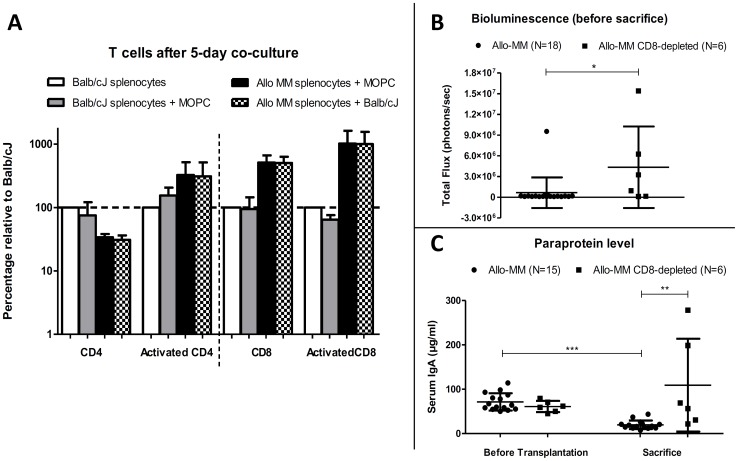
Involvement of CD8 T cells in the graft-versus-myeloma effect. (**A**) *In vitro* T-cell reactivity. Relative percentages (mean±SD), normalized to those of non-activated Balb/cJ splenocytes alone, are represented for 3 independent experiments of 5-day co-cultures of splenocytes (Balb/cJ alone; Balb/cJ + irradiated MOPC315.BM cells; Allo−MM + irradiated MOPC315.BM cells; or Allo−MM + irradiated Balb/cJ splenocytes). Activated cells displayed a CD69^+^ phenotype. *In vivo* implication of CD8 T cells (CD8 T-cell-depletion of the graft). (**B**) Bioluminescence quantitation. Total flux (photons/sec) measured on the dorsal side just before sacrifice (mean±SD), *p<0.05. (**C**) Paraprotein level. Serum IgA quantitation (µg/ml) by ELISA before transplantation and at sacrifice (mean±SD), **p<0.005; ***p<0.0001 (Mann-Whitney Test).

Based on the previous results suggesting a possible *in vivo* implication of T cells in the GvM effect, we evaluated T-cell reactivity against myeloma or allogeneic cells *in vitro*. We performed a five-day co-culture of splenocytes from Allo-MM mice, with irradiated target cells (MOPC315.BM or BALB/cJ splenocytes). The results showed an expansion of CD8 T cells, in contrast to CD4 T cells, but the expansion observed in co-cultures with MOPC315.BM cells was not different from co-cultures with Balb/cJ splenocytes, suggesting a close relationship between epitopes recognized in chronic GvHD and GvM processes. Furthermore, increased percentages of activated CD4, and even more so activated CD8 T cells, confirmed T-cell reactivity against myeloma cells and Balb/cJ cells among Allo-MM splenocytes (Fig.3 A).

Our previous results suggested reactivity of CD8 T cells against MM. Moreover, MOPC315.BM cells express MHC I, but do not express MHC II, possibly implicating a direct activation of myeloma-reactive CD8 T cells, whereas CD4 T cells need antigen-presenting cells to become reactive against myeloma cells, as previously described [26]. Thus, we evaluated the contribution of CD8 T cells in the GvM effect *in vivo*, by depleting CD8 T cells in the B10.D2 graft before transplantation to myeloma-bearing Balb/cJ mice. CD8 T-cell and activated CD8 T-cell reconstitution was significantly slower in the depleted group 1 week after transplantation, and a trend for lower CD8 T-cell numbers was still noted 3 weeks after transplantation. In the CD8 T-cell-depleted group, 4 out of 6 mice (66.7%) showed strong bioluminescence signals and myeloma symptoms after transplantation, whereas in the standard Allo-MM group a bioluminescence signal was only observed in one out of 18 mice after transplantation (5.6%, p<0.01). Higher tumor burden and paraprotein levels in CD8 T-cell-depleted mice (Fig.3 B-C) confirmed a reduced GvM effect compared to standard allogeneic transplantation, underlining the *in vivo* importance of CD8 T cells in GvM effects.

### Graft-versus-myeloma and graft-versus-host reactivity

**Figure 4:**
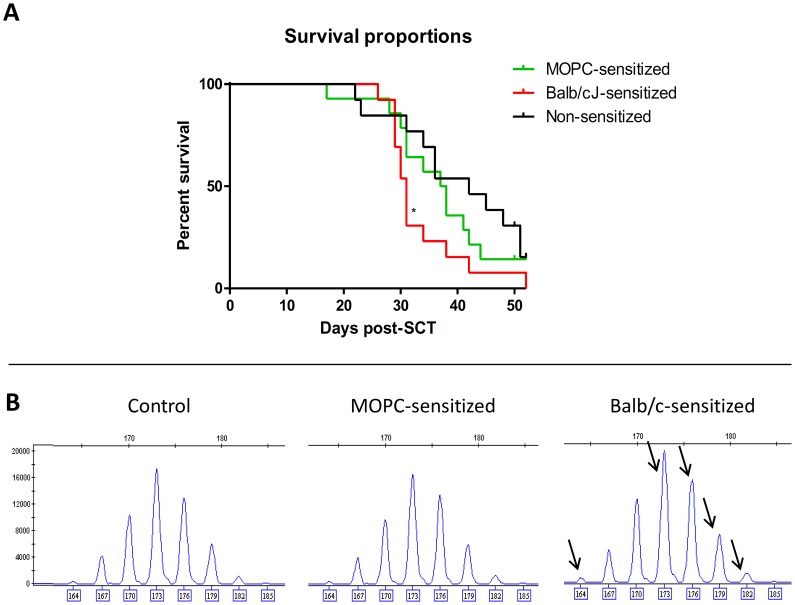
Graft-versus-myeloma and graft-versus-host reactivity. B10.D2 donor mice were not sensitized (”controls”) or previously sensitized by injection of MOPC315.BM myeloma cells or Balb/cJ splenocytes (“MOPC-sensitized” or “Balb/c-sensitized”, respectively). (**A**) Survival proportions of Balb/cJ recipient mice (N = 13–14/group) that were irradiated and received a graft from non-sensitized, “MOPC-sensitized” or “Balb/c-sensitized” B10.D2 mice. Recipient mice were sacrificed when severe GvHD symptoms (GvHD score ≥ 8/10) or apathy were present. *p = 0.038 (Log-Rank Test). (**B**) Representative Vβ15 spectratype histograms for CD4 T cells isolated from splenocytes of non-sensitized B10.D2 mice ( = control), “MOPC-sensitized” or “Balb/c-sensitized” B10.D2 mice. Arrows indicate skewed peaks (i.e. mean peak area> mean+3SD of corresponding control peak).

The separation of GvT effects from unwanted GvHD remains an important challenge in transplantation medicine. In order to determine whether epitopes were shared between immune responses directed against MOPC315.BM myeloma cells and GvH response in our model, we decided to perform sensitization of B10.D2 donor mice by injecting them with myeloma cells prior to cell collection for transplantation. In preliminary experiments (data not shown), Allo-MM recipient mice challenged with myeloma-sensitized donor cells presented with exacerbated chronic GvHD symptoms, indicating possible overlap between myeloma antigens and alloantigens *in vivo.* In order to confirm these results and compare the effects of MM sensitization with allogeneic sensitization, we performed chronic GvHD experiments on larger cohorts, in which B10.D2 donor mice were sensitized with MOPC315.BM myeloma cells (MOPC-sensitized) or Balb/c-splenocytes (Balb/c-sensitized) before transplantation to healthy Balb/c recipient mice (chronic GvHD model). Recipient mice transplanted with grafts from Balb/c-sensitized donors experienced worsened GvHD symptoms and had a shorter survival compared to control animals (Fig.4 A), with a median survival of 31 vs 42 days, respectively (p = 0.038). Notably, flow cytometric results showed reduced percentages of Treg cells and naive T cells (CD4 and CD8) in these mice compared to control mice in spleen, blood and bone marrow at sacrifice (data not shown). Likewise, recipient mice of MOPC-sensitized donor grafts also showed a trend towards shorter survival (median survival 37 days), suggesting that sensitization with myeloma cells could lead to increased alloreactivity in this model.

In order to confirm the possible overlapping responses between GvHD and GvM in our model, we used CDR3-size spectratype analyses to determine which TCR Vβ families were involved in the different B10.D2 T-cell responses [23], [24], [27]. We analysed the TCR Vβ spectratype within both CD4 and CD8 T cells (isolated from MOPC-sensitized or Balb/c-sensitized B10.D2 mice), as our previous results support a role of CD8 T cells in the GvM effect, whereas chronic GvHD in the B10.D2→Balb/c model is mainly dependent on CD4 T cells [28], [29]. Skewed CDR3-size lengths for different Vβ families are summarized in Table 1, and indicate clonal or oligoclonal expansions. Within CD4 T cells, the results revealed 6 myeloma-reactive Vβ families (2, 3, 5.1, 5.2, 8.3, 11) and 5 alloreactive Vβ families (5.1, 5.2, 11, 15, 18). Reactivity of Vβ families 2, 3 and 8.3 was unique to the anti-myeloma response, whereas alloreactivity specifically involved Vβ families 15 (with five skewed CDR3-lenghts, Fig.4 B) and 18. Vβ families 5.1., 5.2 and 11 showed expansion both in the anti-myeloma and allogeneic settings, suggesting overlapping responses of these CD4 T-cell populations. Surprisingly, within CD8 T-cell compartment there were no uniquely expanded Vβ families. We only observed three reactive Vβ families (5.1, 11, 13) skewed in both groups at exactly the same CDR3-size lengths, also suggesting potential overlap between responses to MM and Balb/c antigens in the CD8 T-cell subset.

**Table 1:** Comparison of the skewed CDR3-size lengths of CD4 or CD8 T cells isolated from splenocytes of B10.D2 mice sensitized with myeloma or Balb/cJ cells, compared to non-sensitized B10.D2 control mice (skewed if mean peak area> mean+3SD of corresponding control peak).

	CD4 T cells		CD8 T cells	
Vβ	MOPC-sensitized	Balb/cJ-sensitized	MOPC-sensitized	Balb/cJ-sensitized
**1**	-	-	-	-
**2**	147	-	-	-
**3**	160	-	-	-
**4**	-	-	-	-
**5.1**	*173; 176*	*173; 176*	*161*	*161*
**5.2**	*207*	*218*	-	-
**6**	-	-	-	-
**7**	-	-	-	-
**8.1**	-	-	-	-
**8.2**	-	-	-	-
**8.3**	152	-	-	-
**9**	-	-	-	-
**10**	-	-	-	-
**11**	*144*	*163*	*165*	*165*
**12**	-	-	-	-
**13**	-	-	*176*	*176*
**14**	-	-	-	-
**15**	-	164; 173; 176; 179; 182	-	-
**16**	-	-	-	-
**18**	-	159	-	-
**20**	-	-	-	-

“–”: no skewing; numbers in table indicate a skewed size length (band) within a particular Vβ family, numbers in italic indicate bands skewed in both groups.

Although we did not observe overall aggravation of GvHD symptoms in the larger cohort of mice transplanted with grafts from MOPC-sensitized donors (data not shown), survival of these mice was shorter compared to control group (Fig.4 A). This result suggests that sensitization with myeloma cells could lead to increased alloreactivity in this model due to the presence of shared antigens between both allogeneic Balb/cJ and MOPC315.BM cells, as demonstrated by the presence of overlapping CDR-3 size skewed bands in both CD4 and CD8 T-cell populations (Table 1).

## Discussion

In the current study, we describe a graft-versus-myeloma effect in the context of MHC-matched allogeneic transplantation in myeloma-bearing mice. So far, only two other immunocompetent mouse models of allogeneic transplantation in MM have been described. In the first model, Balb/c mice were intra-peritoneally injected with plasmacytoma-resembling HOPC-1F cells, which present few characteristics of human MM disease, and transplanted with bone marrow and spleen cells of DBA/2 origin [13]. No long-term disease-free survival could be obtained with unmanipulated SCT alone – since idiotype vaccination of donor mice was needed for the GvM effect - and the transplanted mice developed acute GvHD, which has a distinct pathobiology from that of chronic GvHD.

In the other murine MM allo-SCT model [14], C57Bl/KaLwRij.Hsd (H-2^b^) recipient mice first received allo-SCT from MHC-matched C3.SWH2b/SnJ donors. After two months of immune reconstitution, recipients were inoculated with the 5T33MM murine cell line and developed myeloma disease. Donor lymphocyte infusions (DLI) prolonged the median survival of diseased mice. Additional dendritic cell (DC) vaccination of the DLI-recipient mice, using dendritic cells loaded with the H7 minor histocompatibility antigen that differs between donor and recipient strains, further extended survival without inducing GvHD by targeting the H7-presenting MM cells. Percentages of effector memory CD8 T cells were increased in the bone marrow of transplanted MM mice, irrespective of post-transplantation treatment. However, in this GvM model, the observed anti-myeloma effect is entirely due to post-transplantation immunotherapy using DLI, as mice received allo-SCT before the establishment of MM disease, which does not correspond to the clinical scenario.

In our study, we established a GvM model using the MOPC315.BM model, which closely resembles human MM disease as tumor cells mainly grow in the bone marrow milieu and induce osteolytic lesions [18]. A GvM effect was obtained using allo-SCT in mice with established MM disease, as a curative treatment, with concomitant chronic GvHD development. The allogeneic graft was composed of bone marrow (source of hematopoietic stem cells) and splenocytes (source of T cells), which is a widely used approach in murine SCT models [30]. In clinical SCT, the most frequently used graft sources are hematopoietic stem cells collected from peripheral blood after a mobilization treatment [31]. These grafts contain 10 to 30-fold higher amounts of T cells and other immune cells (B cells, monocytes, NK and NKT cells) than bone marrow grafts [32]. Thus, murine SCT grafts that contain bone marrow enriched with splenocytes (predominantly consisting in B and T cells, monocytes, NK and NKT cells) display similarities in cellular composition with peripheral blood-derived grafts used in clinics. Even though some dissimilarities may exist between murine SCT protocols and clinical allo-SCT, such murine models are invaluable tools in understanding the immunobiology of SCT [30].

Our data corroborate the role of T cells in the observed GvM effect. In general, both CD4 and CD8 T-cell subsets contribute to graft-versus-leukemia (GvL) reactions. However, the dominant mechanism seems to be strain-specific and varies with the degree of donor-recipient histocompatibility [33]. Mice receiving CD8-depleted donor marrow have a higher leukemic relapse incidence than those receiving CD4-depleted marrow [34], [35]. In experimental mouse transplants, the addition of purified CD8 T cells to the graft had an anti-tumor effect and facilitated engraftment without inducing GvHD [36]. In our model, *in vivo* and *in vitro* data suggest a role for CD8 T cells in the GvM effect, since CD8 T-cell-depletion of the graft reduced GvM effects (Fig.3 B-C). We identified three TCR Vβ families within the CD8 T-cell subset, with overlapping reactivity to both myeloma and alloantigens. CD8 T cells can recognize polymorphic peptides derived from non-MHC proteins (i.e. minor histocompatiblity antigens) [37], [38]. Thus, we hypothesise that Balb/cJ minor histocompatibility antigens implicated in GvHD pathogenesis are present on MOPC315.BM cells (originating from a Balb/c-derived background). B10.D2 and Balb/cJ mice, both H-2^d^, differ at multiple non-MHC loci (including H-1, H-7, H-8, H-9 and H-13) [39] potentially implicated in alloreactivity and possibly expressed by MOPC315.BM cells, which could explain that the same CDR3-size length was found skewed in both the anti-tumor and the alloresponse (band 161 in Vβ family 5.1, band 165 in Vβ family 11 and band 176 in Vβ family 13, Table 1).

CD4 T cells, which are essential for the development of chronic GvHD in the B10.D2→Balb/c model [28], [29], also probably played a role in the GvM effect. Indeed, we also identified potentially overlapping TCR Vβ families within CD4 T cells (i.e. Vβ families 5.1, 5.2 and 11; Table 1), further confirming a link between GvM and GvHD. Interestingly, we identified other Vβ families within the CD4 T-cell subset that are probably implicated specifically in either GvM or GvH effects, suggesting that GvM or GvH reactivity could be separately modulated in this model in future studies. Despite the lack of MHC II expression on MOPC315.BM cells, primary CD4 T-cell responses (mediated by tumor-infiltrating antigen-presenting cells) can be induced, as demonstrated for the MHC II-negative parental MOPC315 cells [26], [40]. CD4 cells probably play a role in the orchestration of the CD8 T-cell response, and in the establishment of GvHD, as previously described [28]. We did not observe complete disappearance of GvHD after CD8 T-cell-depletion (in mice sacrificed after day 21), most likely because of the presence of alloreactive T cells in the CD4 compartment (as suggested by the presence of single and multiple skewed CDR3-size lengths in the CD4 anti-Balb/cJ response of the Vβ 5.1, 5.2, 11, 15 and 18 families, Table 1 & Fig.4 B) confirming the essential role of CD4 T cells for GvHD development in this model. For future studies using this model, it would be interesting to isolate and infuse T cells from Vβ families that are specifically involved in the GvM effect (Vβ 2, 3 and 8.3) in order to determine the effects on GvHD development and the capacity of these T cells to maintain a GvM effect or, in contrast, to deplete from the graft Vβ families specifically involved in the GvH response (Vβ 15 and 18) and determine the persistence of GvM and GvH effects. Similar experiments have already been described in the literature. The Vβ13 family was shown to be highly skewed in the B10.BR CD8 T-cell response against a myeloid leukemia cell line (MMC6), but not in the alloresponse against CBA recipient mice. Transplantation of low doses of CD8^+^Vβ13^+^ T cells, isolated by magnetic cell separation, induced a slight GvT response with no concomitant acute GvHD development [24]. In the B6→Balb.b (MHC-matched) GvHD response, several Vβ families have been found to be skewed within CD4 T cells. Whereas transplantation of the positively selected skewed Vβ families induced lethal GvHD, mice that received skewed Vβ-depleted CD4 T cells all survived with minimal GvHD symptoms [41].

In conclusion, we describe the establishment of a reliable graft-versus-myeloma model using allo-SCT in immunocompetent tumor-bearing mice. Effector memory CD4 and CD8 T cells probably mediated the GvM effect in this model, with CD8 T cells being essential for the observed GvM effect *in vivo*. Within CD4 and CD8 subsets, we identified overlapping Vβ families in the responses against myeloma cells (anti-tumor) and Balb/cJ cells (alloreactive), underlining the relationship between anti-tumor responses and GvHD, whereas some Vβ families within CD4 T cells specifically respond to either myeloma or host alloantigens. The current murine model of GvM should enable future studies of immunomodulatory drugs, acting on the balance between graft-versus-myeloma and graft-versus-host effects.
