# Correction: Establishment of a Murine Graft-versus-Myeloma Model Using Allogeneic Stem Cell Transplantation

**DOI:** 10.1371/journal.pone.0117643

**Published:** 2015-02-17

**Authors:** The PLOS ONE Staff

There are errors in the Author Contributions section. The correct contributions are: Conceived and designed the experiments: MB LB FB JC. Performed the experiments: MB EO LB. Analyzed the data: MB YB BB JZ FB JC. Contributed reagents/materials/analysis tools: BB JZ PD. Wrote the paper: MB YB MH AB RH FB JC.

## References

[1] Binsfeld M, Beguin Y, Belle L, Otjacques E, Hannon M, et al (2014) Establishment of a Murine Graft-versus-Myeloma Model Using Allogeneic Stem Cell Transplantation. PLoS ONE 9(11): e113764 doi: 10.1371/journal.pone.0113764 25415267 10.1371/journal.pone.0113764 PMC4240591

